# Attenuation of a Highly Pathogenic Porcine Deltacoronavirus Strain CZ2020 by a Serial Passage *In Vitro*

**DOI:** 10.1155/2023/2830485

**Published:** 2023-03-31

**Authors:** Wenlong He, Qi Peng, Jizong Li, Jin Huang, Xuhang Cai, Siyuan Li, Baotai Zhang, Li Xiao, Jie Gao, Chuanhong Wang, Jiali Qian, Laqiang Gu, Rui Wang, Xuechao Tang, Kemang Li, Xu Song, Jinzhu Zhou, Mingjun Zhu, Bin Li

**Affiliations:** ^1^College of Veterinary Medicine, Hebei Agricultural University, Baoding 071001, China; ^2^Nanchang City Key Laboratory of Animal Virus and Genetic Engineering, Nanchang 330045, China; ^3^College of Bioscience and Engineering, Jiangxi Agricultural University, Nanchang 330045, China; ^4^Institute of Veterinary Medicine, Jiangsu Academy of Agricultural Sciences, Key Laboratory of Veterinary Biological Engineering and Technology, Ministry of Agriculture, Nanjing 210014, China; ^5^Jiangsu Key Laboratory for Food Quality and Safety-State Key Laboratory Cultivation Base, Ministry of Science and Technology, Nanjing 210014, China; ^6^Jiangsu Co-Innovation Center for the Prevention and Control of Important Animal Infectious Diseases and Zoonoses, Jiangsu Key Laboratory of Zoonoses, Yangzhou University, Yangzhou 225009, China; ^7^College of Veterinary Medicine, Guizhou University, Guiyang 550025, China

## Abstract

Porcine deltacoronavirus (PDCoV) is an emerging swine coronavirus that causes severe diarrhea to pigs of all ages, especially the suckling piglets under one-week-old. We previously isolated a highly pathogenic PDCoV strain, CZ2020, from a diarrheal piglet and have passaged it for over 100 passages. The adaptability of the CZ2020 increased gradually *in vitro* as the passage increased. Amino acid mutations were observed in pp1a, pp1ab, spike, envelop, and membrane proteins, and the spike protein accounts for 66.7% of all amino acid mutations. Then, the high passage strains, CZ2020-F80 and CZ2020-F100, were selected for evaluation of the pathogenicity in three-day-old piglets to examine whether these amino acid changes affected their virulence. At 2 days postchallenge (DPC), 2/5 piglets started to show typical diarrhea, and at 4 DPC, severe diarrhea was observed in the CZ2020-challenged piglets. Viral RNA could be detected at 1 DPC in rectal swabs and reached its highest at 4 DPC in the CZ2020-challenged group. CZ2020-F80- and CZ2020-F100-challenged groups have one piglet exhibiting mild diarrhea at 4 and 6 DPC, respectively. Compared with the CZ2020-challenged group, the piglets in CZ2020-F80- and F100-challenged groups had lower viral loads in rectal swabs, intestines, and other organs. No obvious histopathological lesions were observed in the intestines of CZ2020-F80- and F100-challenged piglets. Virulent PDCoV infection could also induce strong interferons and proinflammatory cytokines *in vitro* and *in vivo*. These data indicate that the strains, CZ2020-F80 and CZ2020-F100, were significantly attenuated via serial passaging *in vitro* and have the potential for developing attenuated vaccine candidates.

## 1. Introduction

The family *Coronaviridae* can be genetically divided into four genera, including *alphacoronavirus*, *betacoronavirus*, *gammacoroanvirus*, and *deltacoronavirus*. Porcine deltacoronavirus (PDCoV), a member of the genus *deltacoronavirus*, was first identified in a surveillance study in Hong Kong in 2012 [1]. In February 2014, PDCoV was detected in diarrheal swine in the USA and rapidly spread over 20 states in the USA [2]. PDCoV has subsequently been detected in many other pig-raising countries, including Mainland China [3], Canada [4], South Korea [5], Japan [6], Thailand, Laos, and Vietnam [7]. As an emerging swine coronavirus, PDCoV can cause mild to severe diarrhea in pigs of all ages, especially in suckling piglets, which results in huge economic losses to the pig industry [8, 9]. However, there are no effective drugs and vaccines available to control and prevent PDCoV diseases currently. Moreover, this emerging agent also has great potential for cross-species transmission [10]. Recently, PDCoV infection in children with acute febrile illness was reported in Haiti which indicates the risk of PDCoV transmission among the human population [11].

PDCoV is an enveloped, single-stranded, positive-sense RNA virus with a genome of approximately 25.4 kb in length. The 5′ two-thirds of the genome contain two open reading frames (ORFs), ORF1a and ORF1b, encoding two overlapping polyproteins pp1a and pp1ab which are further proteolytically processed into 15 nonstructural proteins (nsp2–nsp16). The remaining one-third encoding structural and accessory proteins are in the following order: spike (S), envelop (E), membrane (M), NS6, nucleocapsid (N), and NS7/NS7a (located within N gene) proteins [12]. The *S* protein is responsible for binding to specific host receptors for initiating the cell entry of the virus. It is also a good antigen for efficiently inducing neutralization antibodies, which are often used for the development of subunit vaccines. E, M, and N proteins are the most conserved proteins among the structural proteins, which are often employed as the target for design diagnostic tools. NS6 can inhibit type I interferon production and is an important virulence factor of PDCoV [13, 14]. NS7 is a nonessential protein for PDCoV replication and is extensively distributed in the mitochondria of a cell line expressing NS7 protein [14, 15].

Currently, PDCoV has existed in most pig-raising countries and continues to cause economic losses in the pig industry. Together with strict biosecurity measures, immunization with vaccines is an important method to prevent and control PDCoV diseases. Because PDCoV mainly infects the epithelial cells of the piglet's small intestines, mucosal immunity, especially the secretory IgA (sIgA), plays an important role in the clearance process of PDCoV. In general, live attenuated vaccines tend to elicit higher levels of sIgA than inactivated vaccines, DNA vaccines, and subunit vaccines. To date, no vaccines and drugs are available for the treatment and prevention of PDCoV diseases; so, there is a great urgency to develop live-attenuated virus vaccines.

In this study, a virulent PDCoV strain, CZ2020, isolated in our laboratory in 2020, was propagated *in vitro* for over 100 passages. The biological characteristics of CZ2020 and its high passages were characterized, and we also identified the genomic changes during serial passaging. Finally, the pathogenicity and host immune responses of the original CZ2020 and the high passages (passages 80 and 100) were evaluated in a three-day-old piglet model.

## 2. Materials and Methods

### 2.1. Cell Line, Viruses, and Antibodies

The LLC-PK1 cell line was cultured in DMEM containing 10% fetal bovine serum (Tianhang Biotech, Hangzhou, China) at 37°C with 5% CO_2_. PDCoV strains, CZ2020, CZ2020-F80, and CZ2020-F100, were propagated in LLC-PK1 cells with 7.5 *μ*g/ml trypsin (Sigma). The PDCoV strains CZ2020-F80 and CZ2020-F100 are the passage of the 80^th^ and 100^th^ strains of CZ2020, respectively. Pig anti-PDCoV polyclonal antibody was produced in our laboratory and was well characterized [16]. Goat anti-pig IgG H&L (FITC) (ab6911) was purchased from Abcam.

### 2.2. Passaging of PDCoV CZ2020 in LLC-PK1 Cells

LLC-PK1 cell monolayer in a T25 cell culture flask was washed twice with PBS and then inoculated with 0.1 MOI of PDCoV. After the incubation of the flask at 37°C for 1.5 h, the cells were rinsed with PBS, and 5 ml of DMEM containing 7.5 *μ*g/ml trypsin was added. The cell culture flask was placed in a 37°C cell culture incubator with 5% CO_2_. When 90% of the cells developed visible cytopathic effects, the samples were freeze-thawed one time and centrifuged at 9000 ×g for 1 min at 4°C to remove the cell debris. The virus pool was used for the inoculation of the cell monolayers for next time use.

### 2.3. Growth Kinetics of Viruses in LLC-PK1 Cells *In Vitro*

Monolayers of LLC-PK1 cells in 6-well plates were washed twice with PBS and then inoculated with PDCoV CZ2020, CZ2020-F80, and CZ2020-F100 at an MOI of 0.01 at 37°C for 1.5 h; then, the plates were washed with PBS for two times and added with 2 ml of DMEM containing 7.5 *μ*g/ml trypsin. The cell culture supernatants collected at indicated time points were subjected to titration of the viral infection by TCID_50_. The growth curves were drawn based on the infectious titers of each virus.

### 2.4. Immunofluorescence Assay

LLC-PK1 cells were seeded into 24-well plates and then infected with 0.1 MOI of PDCoV CZ2020, CZ2020-F80, and CZ2020-F100, respectively. At 10 hours postinfection (hpi), the plates were rinsed twice with PBS and fixed with 4% paraformaldehyde for 15 min. Then, the cells were washed with PBS three times and permeabilized with cold methanol for 10 min. The plates were incubated with 5% skimmed milk at 37°C for 1 h. After washing twice with PBS, the cells were incubated with 1 : 500 diluted pig anti-PDCoV polyclonal antibodies at 37°C for 1 h. Afterwards, the cells were washed with PBS three times and then incubated with FITC-conjugated goat anti-pig IgG H&L at a dilution of 1 : 500 for 1 h at 37°C. The cell nuclei were stained with 0.01% 4′, 6-diamidino-2-phenylindole (DAPI). After washing three times with PBS, the cells were used for fluorescent observation with a fluorescence microscope (Nikon).

### 2.5. Viral Plaque Assay

Monolayers of LLC-PK1 cells in 6-well plates were incubated with 500 *μ*l of 10-fold serially diluted viral stocks at 37°C for 1.5 h. Then, the plates were rinsed two times with PBS and pipetted with 2 ml of DMEM containing 1.5% methylcellulose, 5 *μ*g/ml trypsin, and 37.5 *μ*g/ml pancreatin (Sigma). At 48 hpi, the cells were fixed with 4% paraformaldehyde and stained with 0.1% crystal violet (Sigma).

### 2.6. Sequence Analysis

The complete genome sequences of PDCoV CZ2020, CZ2020-F80, and CZ2020-F100 were determined as previously described [17]. The GenBank accession numbers for PDCoV CZ2020, CZ2020-F80, and CZ2020-F100 were OK546242, ON936274, and ON936275, respectively. Sequence alignment was performed using the ClustalW method in MEGA 7.0 software. The phylogenetic trees were constructed in MEGA 7.0 using the neighbor-joining method with 1000 bootstrap replicates.

### 2.7. Animal Experimental Design

All animal-related experiment protocol was approved by the Jiangsu Academy of Agricultural Sciences Institutional Animal Care and Use Committee (NKYVET 2020-0145). All the procedures in this study involving the piglets were performed in accordance with relevant guidelines and regulations. PDCoV CZ2020, CZ2020-F80, and CZ2020-F100 were selected for evaluation of the pathogenicity in piglets. A total of 20 two-day-old ternary hybrid piglets farrowed from sows free of neutralizing antibodies against PDCoV were confirmed to be negative for PDCoV by RT-PCR. The piglets were randomly allocated into four experimental groups with five piglets per group, and each group was kept in a separate room. At the age of three-day-old, the piglets were orally inoculated with 2  × 10^5^ TCID_50_ of the different passages of PDCoV (original CZ2020, CZ2020-F80, and CZ2020-F100). After inoculation, the clinical sign was observed four times every day and rectal swabs were collected daily for the quantification of virus shedding. Fecal consistency was scored as the following criteria: 0 = solid feces, 1 = pasty feces, 2 = semiliquid feces, 3 = liquid feces, respectively. At the end of the animal experiment, the piglets were euthanized, and the intestinal segments were collected for the determination of the virus distribution and fixed in 10% neutral-buffered paraformaldehyde for histopathology analysis.

### 2.8. Histopathology Analysis

At necropsy, the intestinal segments, including the duodenum, jejunum, ileum, cecum, colon, and rectum, were collected and fixed in 10% neutral-buffered paraformaldehyde. At 48 h of fixation, the intestines were trimmed, processed, and embedded in paraffin. The embedded tissues were cut and routinely stained with hematoxylin and eosin.

### 2.9. Quantitative Real-Time PCR

The total RNA was extracted from rectal swabs and intestinal segments and transcribed into cDNA using a reverse transcription kit (Vazyme Biotech, Nanjing, China). TaqMan-based quantitative real-time PCR was performed to quantify PDCoV N gene transcripts using Taq Pro HS Probe Master mix (Vazyme, Nanjing, China). The cytokines from the cell culture samples and the ileum samples were quantified using the SYBR green PCR master mix (Vazyme Biotech, Nanjing, China). The primer sets for the quantification of PDCoV N and cytokine genes are shown in Table 1.

## 3. Results

### 3.1. Biological Characteristics of PDCoV CZ2020 and Its High Passages

**Figure 1:**
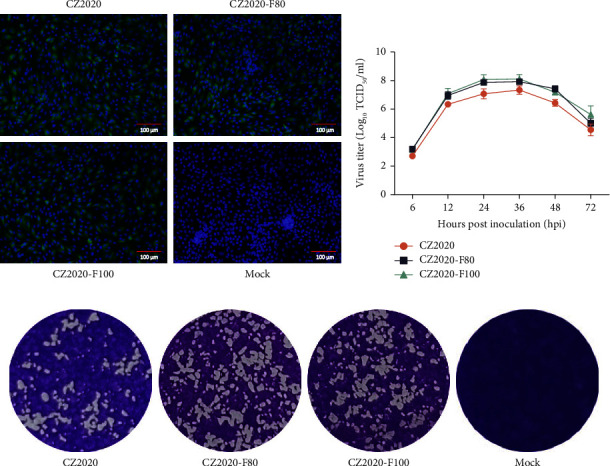
Biological characterization of PDCoV CZ2020 and its high passages, CZ2020-F80 and CZ2020-F100. (a) LLC-PK1 cells were infected with PDCoV CZ2020, CZ2020-F80, and CZ2020-F100 for 12 h; then, PDCoV was detected with immunofluorescence assay (IFA). Porcine polyclonal antibodies were used to detect PDCoV (green), and cell nuclei were stained with DAPI (blue). Scale bar, 100 *μ*m. (b) LLC-PK1 cells were infected with PDCoV strains at an MOI of 0.1. The cell culture supernatants were collected at the indicated time points (6, 12, 24, 36, 48, and 72 hpi). Growth curves were drawn based on the viral titers determined by the TCID50 assay in LLC-PK1 cells. (c) Monolayers of LLC-PK1 cells were infected with PDCoV CZ2020, CZ2020-F80, and CZ2020-F100 for 3 days; then, the plaques were stained with 0.1% crystal violet.

PDCoV strain CZ2020 was isolated from a diarrheal piglet in China in 2020. The serial passaging of PDCoV CZ2020 was carried out in LLC-PK1 cells; then, the passages CZ2020, CZ2020-F80, and CZ2020-F100 were chosen to evaluate the biological characteristics. LLC-PK1 cells infected with PDCoV CZ2020, CZ2020-F80, and CZ2020-F100 showed specific green fluorescence when treated with porcine anti-PDCoV polyclonal antibody, but no green fluorescence was observed in uninfected cells (Figure 1(a)). The multistep growth kinetics of PDCoV strains indicates the three PDCoV strains shared similar growth kinetics in the LLC-PK1 cell culture, which had the highest infectious titer at 36 hpi. However, the infectious titers for CZ2020-F80 and CZ2020-F100 were 10^7.91±0.144^ TCID_50_/ml and 10^8.11±0.314^ TCID_50_/ml, respectively, which are higher than the original PDCoV strain CZ2020 (10^7.3±0.297^ TCID_50_/ml) (Figure 1(b)). These three PDCoV strains also could form plaques with irregular shapes in LLC-PK1 cells, but no significant difference in the plaque size among these three PDCoV strains (Figure 1(c)). In summary, these results indicate that PDCoV CZ2020 gradually increases its adaptability to LLC-PK1 cells as the passage increases.

### 3.2. Genetic Variation Analysis of PDCoV CZ2020 during *In Vitro* Passaging

**Figure 2:**
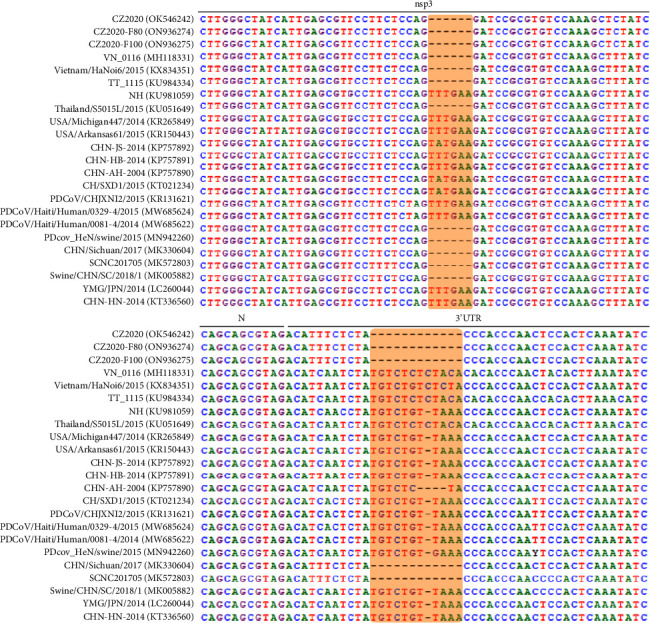
Sequence comparison between CZ2020 and the reference PDCoV strains. CZ2020 variants at different passages and other PDCoV strains were aligned using Clustal W. The dashed lines show the nucleotide deletions in the shaded region.

**Figure 3:**
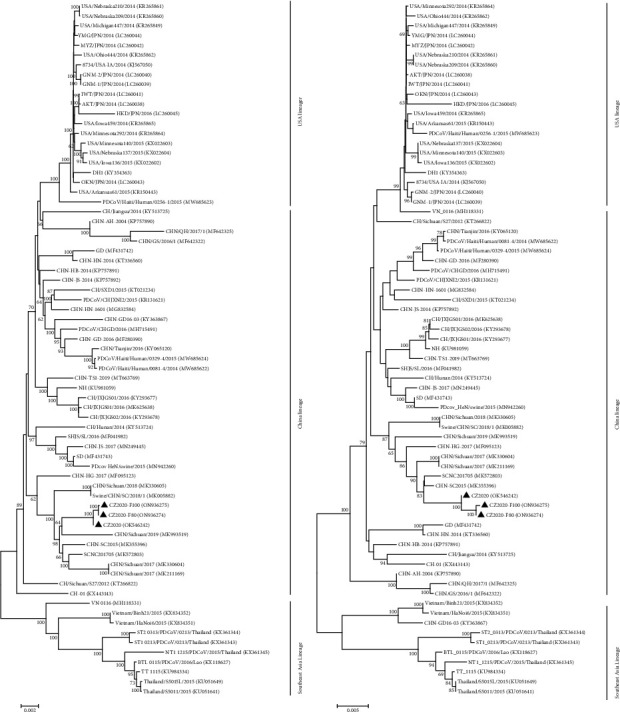
Phylogenetic analysis of PDCoV strains CZ2020 along with other 67 reference PDCoV strains. The trees were constructed using the distance-based neighbor-joining method of the software MEGA 7.0 with 1000 bootstrap replicates, and the GenBank numbers for the reference PDCoVs are shown in the figure. (a, b) The trees were constructed based on the complete genome sequences (a) and the complete spike genes (b). The strains determined in this study were marked with solid black triangles. Scale bars indicate nucleotide substitutions per site.

To analyze the genetic variations of PDCoV CZ2020 variants, the complete genome sequences of the selected PDCoV strains (CZ2020, CZ2020-F80, and CZ2020-F100) were determined and aligned with Clustal W in MEGA 7.0. Compared with PDCoV CZ2020, CZ2020-F80 and PDCoV-F100 had 14 and 17 nucleotide changes which result in 12 and 15 amino acid (aa) changes, respectively (Table 2). The aa changes were mostly located in spike protein, which accounts for 66.7% of total mutations. Partial aa mutations were also observed in other ORFs, including ORF1a, ORF1b, M, and E, which account for 6.7%, 13.3%, 6.7%, and 6.7%, respectively. When aligned with reference PDCoV strains, the CZ2020 and other strains from Sichuan province had a 6-nucleotide deletion in the nsp3 and a 12-nucleotide deletion in 3′UTR (Figure 2). The nucleotide identities of PDCoV CZ2020 with other refence PDCoV strains were 97.1–99.3%. Phylogenetic trees were constructed using the neighbor-joining method with a bootstrap of 1000, and the bootstrap values over 60 are indicated adjacent to the branching points. Phylogenetic analysis of both complete genome and spike genes revealed that the PDCoV strains can be divided into USA, China, and Southeast Asia Lineage, which CZ2020 variants were grouped with PDCoV strains in Sichuan province, which indicates that PDCoV CZ2020 might spread from Sichuan province (Figure 3).

### 3.3. Clinical Sign and Fecal Virus Shedding in Piglets

**Figure 4:**
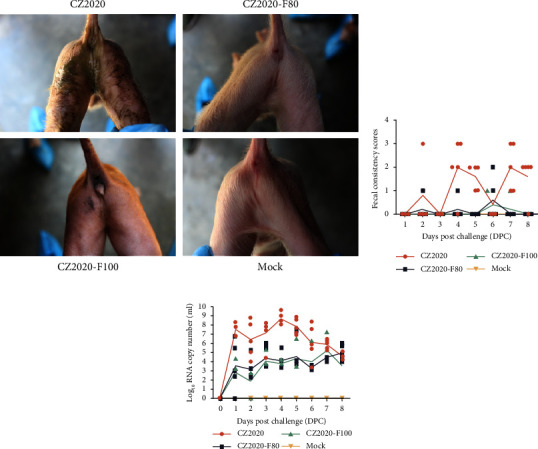
Clinical signs, diarrhea scoring, and fecal viral shedding analyses of the piglets inoculated with PDCoV CZ2020 and its high passages. (a) Representative images of three-day-old pigs challenged or mock-challenged with indicated PDCoVs. (b) Three-day-old piglets were orally inoculated with 2 × 10^5^ TCID_50_ of indicated PDCoVs per pig or mock inoculated with DMEM. Fecal consistency was scored as the following criteria: 0 = solid feces, 1 = pasty feces, 2 = semiliquid feces, 3 = liquid feces, respectively. The plot was drawn based on the fecal consistency scores. (c) Rectal swabs were collected daily from each inoculated piglet, and the viral RNA was quantified by TaqMan-based RT-qPCR with primers targeting the N gene.

To confirm the virulence changes during serial passage *in vitro*, PDCoV CZ2020, CZ2020-F80, and CZ2020-F100 were chosen to evaluate the pathogenicity in three-day-old piglets. Piglets were orally inoculated with 2 × 10^5^ TCID_50_ of indicated viruses. All the piglets in the mock-challenged group were active and showed no clinical signs (Figures 4(a) and 4(b)). However, 2/5 piglets in the CZ2020 group started to show diarrhea in 2 days postchallenge (DPC) but recovered in 3 DPC. At 4 and 5 DPC, 4/5 and 5/5 piglets started to show severe diarrhea, respectively. Almost all the piglets in the CZ2020 group showed severe diarrhea at 7-8 DPC (Figures 4(a) and 4(b)). In the CZ2020-F80-challenged group, one piglet showed mild diarrhea symptoms at 4 and 6 DPC, and the remaining piglets showed no clinical signs during the study. In the CZ2020-F100-challenged group, only one piglet displayed a mild diarrhea symptom at 6 DPC, and other piglets in this group were normal during this study (Figure 4(b)).

The virus RNA could be detected from the rectal swabs on the first-day postinoculation in all these three challenged groups, but the virus shedding is lower in attenuated challenged groups than the virulent challenged group (Figure 4(c)). In the CZ2020-challenged group, the highest virus titer was detected at 4 DPC in feces, when most of the piglets displayed severe diarrhea, and then the virus shedding gradually reduced (Figure 4(c)). The viral RNA was 10^3-4^ copies/g in the feces from CZ2020-F80/100-challenged piglets.

### 3.4. Histopathological Lesions and Virus Distribution in Piglets

**Figure 5:**
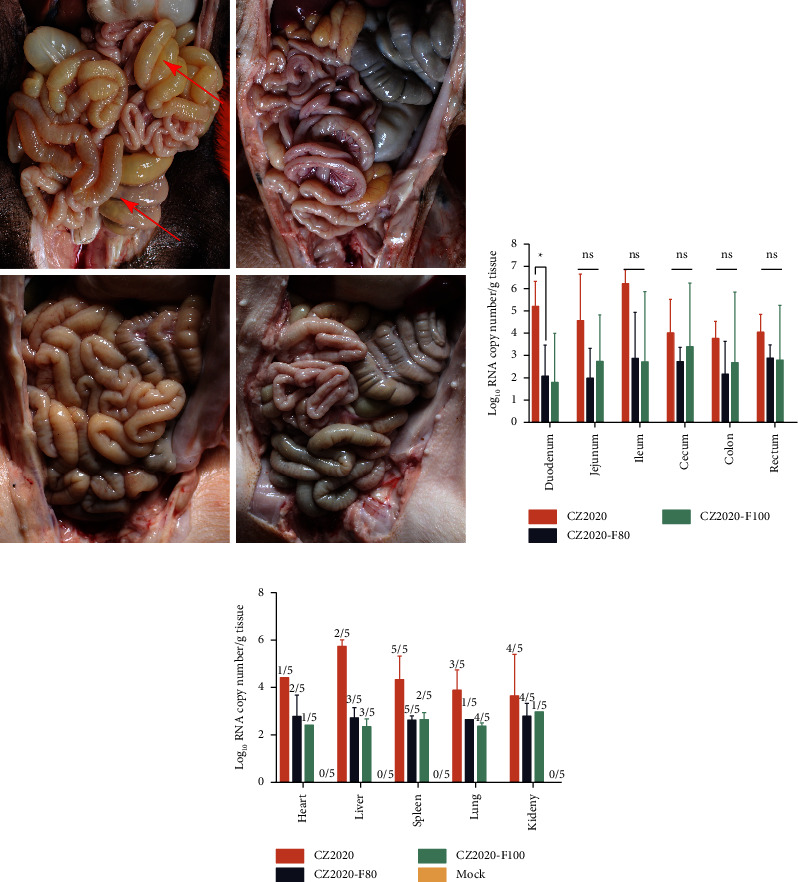
Intestinal lesions of PDCoV-challenged pigs at 8 DPC and virus distribution analysis. (a) Representative images of gross examination of PDCoV-challenged pigs at 8 DPC. The representative intestinal lesions were labeled with red arrows. (b) Three-day-old piglets were orally inoculated with 2 × 10^5^ TCID_50_ of indicated PDCoVs per pig or mock inoculated with DMEM. At 8 DPC, the intestine samples were collected for the determination of the virus load in each intestinal segment by TaqMan-based RT-qPCR with primers targeting the N gene.^*∗*^*P* < 0.05; ns, no significance. (c) Heart, liver, spleen, lung, and kidney samples from challenged piglets at 8 DPC were collected for the determination of the virus load in each intestinal segment by TaqMan-based RT-qPCR with primers targeting the N gene.

Transparent, thin-walled, and gas-distended intestines containing yellow watery contents were observed in CZ2020-challenged piglets at necropsy (8 DPC) that had exhibited severe diarrhea, but no difference between CZ2020-F80/100-challenged piglets and the mock group in gross examination was observed (Figure 5(a)).

**Figure 6:**
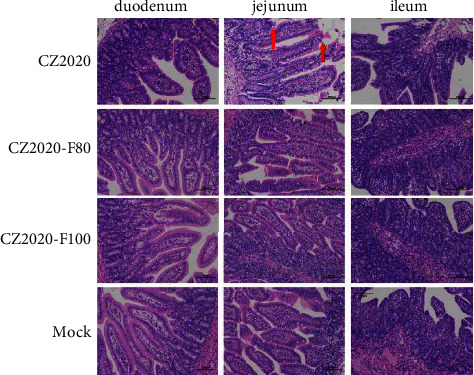
Histopathological lesion analysis of the intestines at 8 DPC. Representative images of the hematoxylin and eosin-stained duodenum, jejunum, and ileum tissue sections of inoculated piglets. Scale bar, 100 *μ*m. The histopathological lesion was labeled with red arrows.

The virus load in different organs of the inoculated piglets was also detected at the end of the animal experiment. The virus could be detected in the intestines (including all the intestinal segments), heart, liver, spleen, lung, and kidney, indicating the broad tissue tropism of PDCoV (Figures 5(b) and 5(c)). Overall, we found a higher virus load in the organs of CZ2020-challenged piglets than in CZ2020-F80/100- challenged piglets (Figures 5(b) and 5(c)). In the CZ2020-challenged group, the viral load in the intestines were 10^5.212±1.112^ copies/g in duodenum, 10^4.572±2.077^ copies/g in jejunum, 10^6.221±0.6303^ copies/g in ileum, 10^4.017±1.501^ copies/g in cecum, 10^3.771±0.7606^ copies/g in colon, and 10^4.051±0.7983^ copies/g in rectum (Figure 5(b)). The virus load in other organs were 1/5 heart (10^4.421^ copies/g), 2/5 liver (mean 10^5.745^ copies/g), 4/5 spleen (mean 10^4.334^ copies/g), 3/5 lung (mean 10^3.889^ copies/g), and 4/5 kidney (mean 10^3.647^ copies/g) (Figure 5(c)). However, the virus load in the heart, liver, spleen, lung, and kidney of the CZ2020-F80/100-challenged groups was lower than the CZ2020-challenged group for 10^1−2^ copies/g. No viral RNA could be detected from the negative control group. Intestinal villous atrophy was observed in the jejunum and ileum of CZ2020-challenged piglets at necropsy (Figure 6). Intestinal vacuolation was obvious in the duodenum and jejunum of CZ2020-challenged piglets when compared with other groups. However, no obvious histopathological lesions were observed in CZ2020-F80/100-challenged groups.

### 3.5. Detection of the Cytokines after CZ2020 Infection

**Figure 7:**
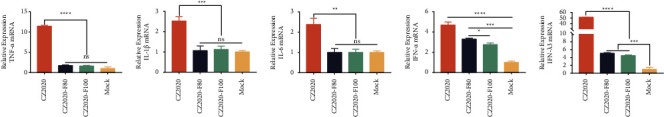
Determination of cytokines after the infection of PDCoV *in vitro*. LLC-PK1 cells were infected or mock infected with PDCoV CZ2020, CZ2020-F80, and CZ2020-F100 for 12 h; then, the infected cells were harvested for quantification of the cytokines. The cytokines, including (a) TNF-*α*, (b) IL-1*β*, (c) IL-6, (d) IFN-*α*, and (e) IFN-*λ*3, were determined by using the SYBR green-based RT-qPCR. The experiments were performed in quadruplicate and data are expressed as the mean ± standard deviations for four samples. An unpaired two-tailed *t*-test was used to determine the statistical significance. ^*∗*^*P* < 0.05; ^*∗∗*^*P* < 0.01; ^*∗∗∗*^*P* < 0.001; ^*∗∗∗∗*^*P* < 0.0001; ns, no significance.

**Figure 8:**
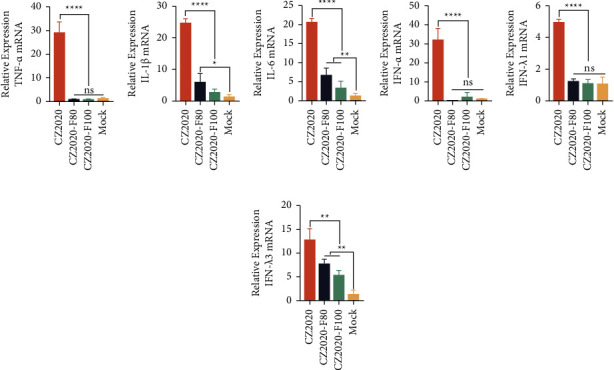
Determination of cytokines in the ileum tissues. The cytokines, including (a) TNF-*α*, (b) IL-1*β*, (c) IL-6, (d) IFN-*α*, (e) IFN-*λ*1, and (f) IFN-*λ*3, in the ileum samples were determined by using the SYBR green-based RT-qPCR. The experiments were performed in triplicate and data are expressed as the mean ± standard deviations for triplicate samples. An unpaired two-tailed *t*-test was used to determine the statistical significance. ^*∗*^*P* < 0.05; ^*∗∗*^*P* < 0.01; ^*∗∗∗∗*^*P* < 0.0001; ns, no significance.

The host cells will produce cytokines in response to pathogen infections. To evaluate the innate immune response to PDCoV infection *in vivo*, we quantify the cytokines after CZ2020 infection *in vitro* and *in vivo*. We found the highly pathogenic CZ2020 infection could induce higher proinflammatory and interferon *in vitro* (Figure 7). The high passage strain could induce higher type I/III interferons LLC-PK1 cells (Figure 7). The *in vivo* studies revealed that compared to the mock-challenged group, the highly pathogenic strain, CZ2020, could induce stronger cytokines including type I/III interferons and proinflammatory cytokines (*P* < 0.05) (Figure 8). Notably, except for that CZ2020-F80 infection induced higher IL-1*β*, IL-6, and IFN-*λ*3 than the control group (*P* < 0.05), there is no significant difference in other cytokines between attenuated infection groups and the mock infection group (Figure 8). These results indicate that pathogenic PDCoV infection could induce cytokines both *in vitro* and *in vivo* in response to viral infection.

## 4. Discussion

Many newly emerged viruses originated from animals, and animals not only served as important protein resources for humans but also have the potential for viral disease emergence [18]. PDCoV was identified from a surveillance study in swine and currently is increasing in prevalence in Southeast Asia and has been detected globally, which caused significant losses to the swine industry. Moreover, human cases with PDCoV infection were reported and PDCoV can use human APN for entry into host cells, indicating the great threat of PDCoV to public health [11, 16]. As a newly emerged swine virus, there are no licensed vaccines and antiviral drugs available for the swine industry. An attenuated vaccine is a promising method for preventing and controlling the disease caused by PDCoV; so, we serially passaged a pathogenic PDCoV strain, CZ2020, which was isolated from Jiangsu, China in 2020 *in vitro* to obtain an attenuated vaccine candidate. Animal experiments suggested the virulence of both CZ2020-F80 and CZ2020-F100 dramatically attenuated. Future experiments will be carried out to evaluate the protective efficacy of the attenuated strains, CZ2020-F80 and CZ2020-F100.

As we propagate the PDCoV *in vitro*, the mutations will accumulate in the genome, especially the spike gene, which is an important antigen for inducing neutralization antibodies. We also detected 17 nucleotide mutations (15 amino acid mutations) during the serial passage *in vitro*, and 10 amino acid mutations were located on the spike protein. Several teams have already reported the major epitopes in the spike protein of PDCoV. Chen et al. reported that the C-terminal domain of the S1 subunit (CTD, aa 278–616) had the most potent PDCoV-neutralizing effect, indicating that CTD of the S1 subunit had major epitopes [19]. Liu et al. found two domains (S1^A^ and S1^B^) in the S1 subunit could elicit potent neutralizing antibodies against PDCoV infection [20]. We found six mutations in CZ2020-F80 and F100 located in S1^A^ and S1^B^ domains, and further experiments are needed to evaluate the protective efficacy of these attenuated strains.

The molecular mechanism for PDCoV attenuation is not fully elucidated yet, and this is the first study to attenuate a virulent PDCoV strain via a serial passage *in vitro*. Based on the heterogenicity analysis of the spike gene, the PDCoV strains from Thailand, Laos, and Vietnam formed a separate lineage [21]. Currently, the pathogenicity of various PDCoV strains was different, and it seems that the Thailand-lineage PDCoV strains showed higher pathogenicity than other lineage strains [22]. Hu et al. passaged PDCoV strain TC-PDCoV OH-FD22 40 times and found that it still was enteropathogenic for gnotobiotic piglets [23]. Our study passaged CZ2020 *in vitro* up to 100 passages, and animal experiments demonstrated that the 80^th^ and 100^th^ were attenuated strains, as evidenced by the severity of clinical signs, histopathological lesions, and the distribution of PDCoV antigens in the gut. When comparing the spike gene with OH-FD22, the OH-FD22 (P40) had only 5 amino acid mutations [23]. We found 10 amino acid mutations in the PDCoV strains, CZ2020-F80 and F100, which accounts for 66.7% of the total amino acid mutations. With the passaging of the virus *in vitro*, the pathogenicity will gradually decrease. Because the spike gene is the main mutant gene during passaging, we hypothesize that the spike gene may be an important virulence gene, but further experiments with the PDCoV reverse genetic tool are needed to prove that conclusion.

Proinflammatory cytokines and interferons are the first line of defense to pathogen infections, especially the type III interferon plays a key role in intestinal mucosal immunity [24]. Previous studies have suggested that PDCoV inhibits type I interferon production *in vitro* [13, 25], but Saeng-Chuto et al. reported that PDCoV infection could upregulate type Iinterferon and proinflammatory cytokine IL12 at 5 DPC [26]. Recently, Wu et al. found PDCoV infection could induce IL-8 production via NF-*κ*B and AP-1 activation by E protein [27]. In consistency with Saeng-Chuto et al. and Wu et al., we also found that both types of I/III interferon and proinflammatory factors were significantly upregulated *in vivo* at 8 DPC (Figure 8). These results suggest that the host activates a series of immune responses after PDCoV infection, but the further molecular mechanism of this phenomenon remains to be elucidated.

In summary, we successfully attenuated the highly pathogenic PDCoV strain CZ2020 via continuing passaging in LLC-PK1 cells and evaluated the pathogenicity of the high passage strains. The results proved that the high passages of PDCoV CZ2020 replicated more efficiently in LLC-PK1 cells but markedly reduced the pathogenicity in three-day-old piglets. Moreover, the highly pathogenic PDCoV strain can induce higher levels of cytokines than the attenuated PDCoV strains.

## Figures and Tables

**Table 1:** Primer sequences used in this study.

Primer ID	Sequence (5′–3′)
PDCoV-NF	TGGCAATGGAGTTCCGCTTA
PDCoV-NR	GGGTATCATTAGGAGGGAGTT
PDCoV-ks-N	FAM-TGGCACAGGTCCCAGAGGAAATCT-BHQ1
IFN-*α*-F	TTCTGCACTGGACTGGATC
IFN-*α*-R	TCTGTGGAAGTATTTCCTCACAG
IFN-*λ*1-F	GGTGTTGGCGACTGTGATG
IFN-*λ*1-R	GATTGGAACTGGCCCATGTG
IFN-*λ*3-F	TTGGCCCAGTTCAAGTCTCT
IFN-*λ*3-R	GAGCTGCAGTTCCAGTCCTC
IL-6-F	CTGGCAGAAAACAACCTGAACC
IL-6-R	TGATTCTCATCAAGCAGGTCTCC
IL-1*β*-F	AACGTGCAGTCTATGGAGT
IL-1*β*-R	GAACACCACTTCTCTCTTCA
TNF-*α*-F	AACCTCAGATAAGCCCGTCG
TNF-*α*-R	ACCACCAGCTGGTTGTCTTT

**Table 2:** Amino acid and nucleotide changes among PDCoV: CZ2020, CZ2020-F80, and CZ2020-F100.

Genomic regions	Nucleotide^a^				Amino acid^b^			
	Position	CZ2020	F80	F100	Position	CZ2020	F80	F100
5'UTR (1–539)	—^c^	—	—	—	—	—	—	—

ORF1a (540–11,408)	6170	C	T	T	1877	—	—	—
	6939	C	T	T	2134	Leu	Phe	Phe
	11,198	T	C	C	3553	—	—	—

ORF1ab (540-11,408, 11,408-19,336)	17,009	G	A	A	5491	Gly	Ser	Ser
	18,423	C	T	T	5962	Ser	Leu	Leu

Spike (19,318–22,797)	19,735	A	A	C	140	Ile	Ile	Leu
	19,796	T	C	C	160	Phe	Ser	Ser
	19,837	C	T	T	174	Leu	Phe	Phe
	19,862	C	T	T	182	Thr	Ile	Ile
	19,894	G	C	C	193	Asp	His	His
	20,379	T	G	G	354	Asn	Lys	Lys
	21,542	G	C	C	742	Gly	Ala	Ala
	21,965	T	A	A	883	Met	Lys	Lys
	22,448	A	G	G	1044	Asn	Ser	Ser
	22,597	C	C	A	1094	Leu	Leu	Ile

Envelope (22,791–23,042)	22,831	A	G	G	14	Tyr	Cys	Cys

Membrane (23,035–23,688)	23,509	G	G	T	159	Asp	Asp	Tyr

NS6 (23,688–23,972)	—	—	—	—	—	—	—	—

Nucleocapsid (23,993–25,021)	—	—	—	—	—	—	—	—

3'UTR (25,022–25,402)	—	—	—	—	—	—	—	—

^a^The nucleotide position was corresponding to the PDCoV CZ2020 strain (GenBank accession No. OK546242). ^b^Synonymous mutations were not shown; the amino acid positions were corresponding to each respective open reading frame. ^c^No nucleotide or amino acid change occurred.

## Data Availability

The datasets used to support the findings of this study are available from the corresponding author on reasonable request.
